# Molecular and Cellular Mechanisms of *Shigella flexneri* Dissemination

**DOI:** 10.3389/fcimb.2016.00029

**Published:** 2016-03-11

**Authors:** Hervé Agaisse

**Affiliations:** Department of Microbiology, Immunology, and Cancer Biology, University of Virginia School of MedicineCharlottesville, VA, USA

**Keywords:** *S. flexneri*, spread from cell to cell, membrane protrusion, double membrane vacuole, IcsA, ARP2/3, N-WASP, type 3 secretion system

## Abstract

The intracellular pathogen Shigella flexneri is the causative agent of bacillary dysentery in humans. The disease is characterized by bacterial invasion of intestinal cells, dissemination within the colonic epithelium through direct spread from cell to cell, and massive inflammation of the intestinal mucosa. Here, we review the mechanisms supporting S. flexneri dissemination. The dissemination process primarily relies on actin assembly at the bacterial pole, which propels the pathogen throughout the cytosol of primary infected cells. Polar actin assembly is supported by polar expression of the bacterial autotransporter family member IcsA, which recruits the N-WASP/ARP2/3 actin assembly machinery. As motile bacteria encounter cell-cell contacts, they form plasma membrane protrusions that project into adjacent cells. In addition to the ARP2/3-dependent actin assembly machinery, protrusion formation relies on formins and myosins. The resolution of protrusions into vacuoles occurs through the collapse of the protrusion neck, leading to the formation of an intermediate membrane-bound compartment termed vacuole-like protrusions (VLPs). VLP formation requires tyrosine kinase and phosphoinositide signaling in protrusions, which relies on the integrity of the bacterial type 3 secretion system (T3SS). The T3SS is also required for escaping double membrane vacuoles through the activity of the T3SS translocases IpaB and IpaC, and the effector proteins VirA and IcsB. Numerous factors supporting envelope biogenesis contribute to IcsA exposure and maintenance at the bacterial pole, including LPS synthesis, membrane proteases, and periplasmic chaperones. Although less characterized, the assembly and function of the T3SS in the context of bacterial dissemination also relies on factors supporting envelope biogenesis. Finally, the dissemination process requires the adaptation of the pathogen to various cellular compartments through transcriptional and post-transcriptional mechanisms.

## Introduction

**Figure 1:**
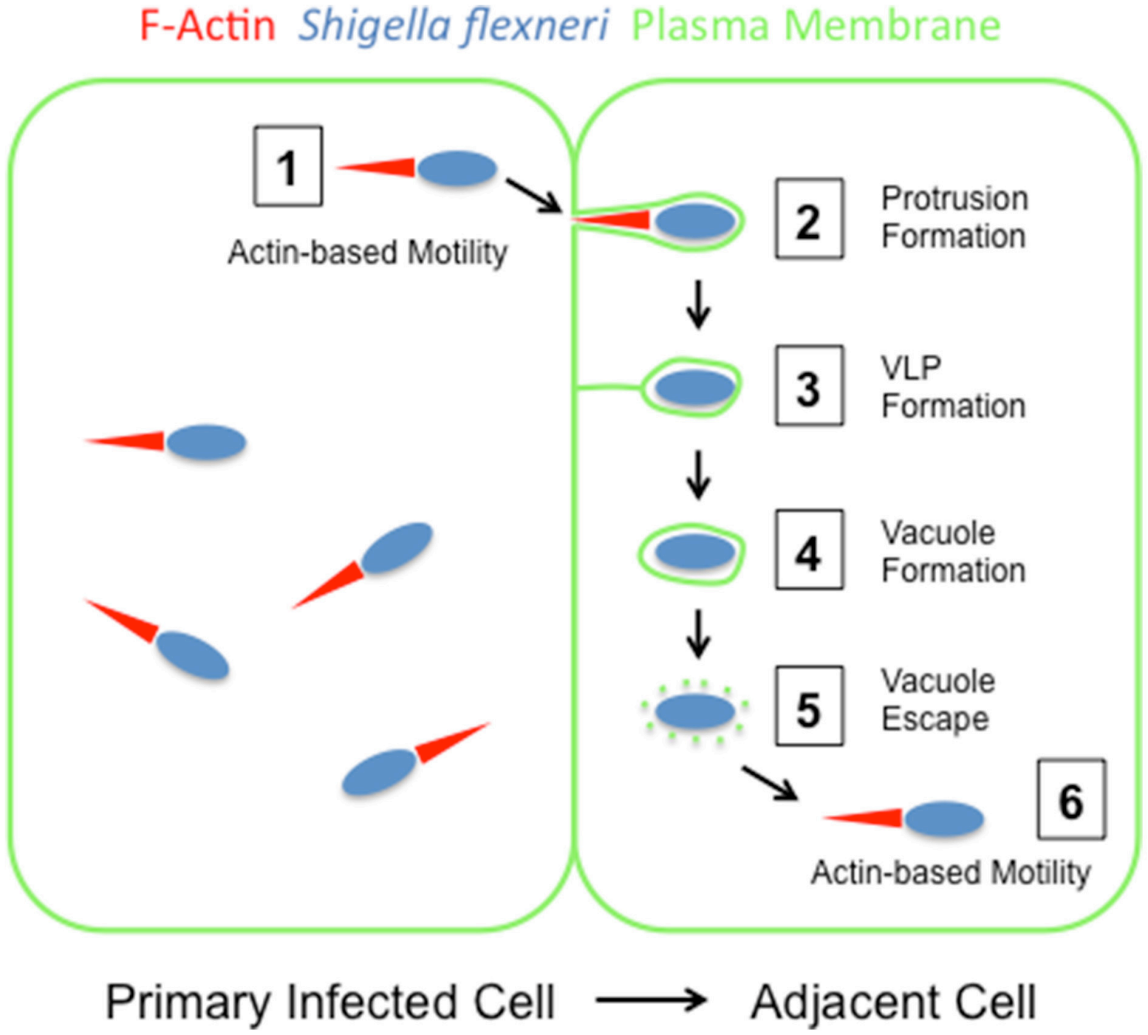
**The multiple steps of ***Shigella flexneri*** dissemination**. *S. flexneri* (blue) invades epithelial cells (plasma membrane, green) and gains access to the cytosolic compartment where bacteria grow and divide (left cell). Step 1: *S. flexneri* acquires actin (red)-based motility in the primary infected cell (left cell), which propels the pathogen throughout the cytosol. Step 2: As motile bacteria encounter cell-cell contacts, they form membrane protrusions that project into the adjacent cell (right cell). Step 3: Membrane protrusions transition into vacuole-like protrusions (VLP) through the collapse of their protrusion neck. Step 4: VLPs resolve into genuine vacuoles that are not connected to the primary infected cell any longer. Step 5: The pathogen escapes the vacuole. Step 6: The pathogen resumes actin-based motility in the adjacent cell.

The intracellular pathogen *Shigella flexneri* is the causative agent of bacillary dysentery in humans (Musher and Musher, 2004). The disease is characterized by bacterial invasion of intestinal cells, dissemination within the colonic epithelium through bacterial spread from cell to cell, and massive inflammation leading to destruction of the epithelial mucosa (Sansonetti, 1998). Spreading defective but otherwise fully invasive bacterial strains are essentially avirulent, highlighting the ability of the bacteria to spread from cell to cell as a central determinant of *S. flexneri* pathogenesis (Makino et al., 1986; Bernardini et al., 1989). The spreading process relies on the acquisition of actin-based motility in the cytosol of primary infected cells (Figure 1, step 1). As motile bacteria encounter cell-cell contacts, they form plasma membrane protrusions that project into adjacent cells (Figure 1, step 2). The formed membrane protrusions then transition into an intermediate membrane-bound compartment termed vacuole-like protrusions (VLP) (Figure 1, step 3), whose resolution leads to the formation of genuine vacuoles (Figure 1, step 4). *S. flexneri* subsequently escapes from the formed double membrane vacuoles (Figure 1, step 5), thereby gaining access to the cytosol of adjacent cells and achieving cell-to-cell spread (Figure 1, step 6). The reiteration of this spreading process supports the propagation of the pathogen within the intestinal epithelium. The formation of membrane protrusions and double membrane vacuoles during infection was first documented in electron microscopy studies using rhesus monkeys as animal models of *S. flexneri* infection (Takeuchi et al., 1968). Further electron microscopy studies in human epithelial cell lines confirmed the formation of protrusions and vacuoles in infected cells, providing suitable *in vitro* systems for investigating the mechanisms supporting *S. flexneri* dissemination (Kadurugamuwa et al., 1991; Gouin et al., 1999). The efficiency of *S. flexneri* dissemination is commonly assessed by using plaque formation as readout of cell-to-cell spread (Oaks et al., 1985). Recently, live fluorescence microscopy was used to capture and quantify the whole sequence of events occurring as single bacteria spread from cell to cell through protrusion and vacuole formation (Dragoi and Agaisse, 2014, 2015). Here, we review three decades of research on the mechanisms supporting *S. flexneri* dissemination.

## Actin-based motility in *S. Flexneri* dissemination

**Figure 2:**
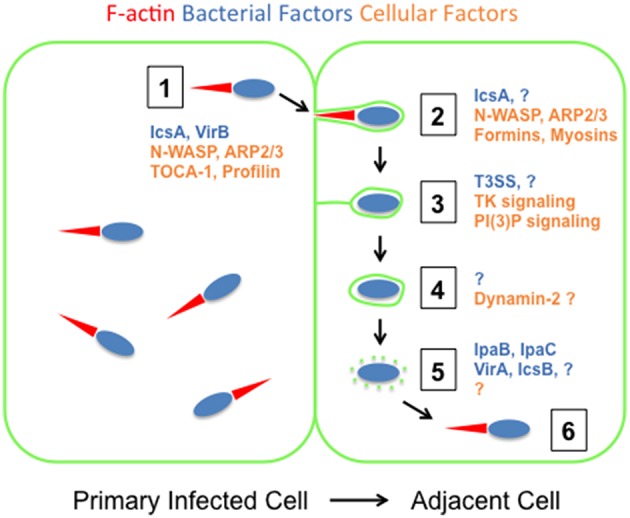
**Bacterial and cellular factors supporting ***Shigella flexneri*** dissemination**. The bacterial and cellular factors involved in *S. flexneri* dissemination are depicted in blue and orange, respectively. Green lines indicate the plasma membrane. Step 1: actin-based motility relies on IcsA that recruits N-WASP, thereby leading to ARP2/3-dependent actin nucleation (red) at the bacterial pole. N-WASP activation is also supported by the VirB-dependent recruitment of TOCA-1. Additional cytoskeleton regulators, such as Profilin stimulate actin-based motility. Step 2: the formation of membrane protrusions is most likely supported by the factors supporting cytosolic motility, including IcsA, N-WASP and the ARP2/3 complex. In addition, formins and myosins are required for efficient protrusion formation. Step 3: membrane protrusions transition into vacuole-like protrusions through a process that rely on the type 3 secretion system (T3SS)-dependent activation of tyrosine kinase and phosphoinositide signaling. Step 4: VLPs transition into genuine vacuoles through resolution of the protrusion neck, a process that potentially relies on the activity of the Dynamin 2-dependent membrane scission machinery in the adjacent cell (right cell). Step 5: the pathogen escapes from the vacuole through a mechanism that requires the activity of the T3SS translocases IpaB and IpaC, as well as the effector protein VirA and IcsB. Step 6: the pathogen resumes actin-based motility through the mechanisms depicted in Step 1.

*S. flexneri* acquires intracellular motility in the cytosol of primary infected cells through manipulation of the host cell actin cytoskeleton (Welch and Way, 2013). The assembly and expansion of the actin network at the bacterial pole propels the pathogen throughout the cytosolic compartment, and leads to the formation of filamentous actin structures referred to as actin tails (Vasselon et al., 1992). Seminal genetic studies led to the identification of the bacterial factor IcsA (aka VirG) as the major determinant of *S. flexneri* actin-based motility (Makino et al., 1986; Bernardini et al., 1989). IcsA is a member of the auto-transporter family of proteins, which display a carboxy-terminal beta domain that forms a beta barrel channel and mediates the transfer of the (passenger) amino-terminal alpha domain across the outer membrane (Henderson and Nataro, 2005). The exposure of the passenger domain of IcsA to the cytosol leads to the recruitment of the nucleation-promoting factor N-WASP (Figure 2, step 1; Suzuki et al., 1998; Egile et al., 1999). In turn, N-WASP recruits and activates a major actin nucleator, the ARP2/3 complex (Figure 2, step 1; Egile et al., 1999; Loisel et al., 1999). In cells, the activity of N-WASP is usually regulated by the small-GTPase Cdc42 (Rohatgi et al., 1999). However, N-WASP recruitment and actin tail assembly at the bacterial pole do not require Cdc42 (Mounier et al., 1999; Shibata et al., 2002). The minimal region of IcsA required for N-WASP recruitment and actin polymerization *in vitro* resides in the passenger domain of IcsA (amino acid residues 56–508; Suzuki et al., 1996). Interestingly, IcsA interacts specifically with N-WASP, but not with other N-WASP/WAVE family members, such as WASP (Suzuki et al., 2002), which explains why *S. flexneri* displays actin-based motility in N-WASP-expressing cells, such as epithelial cells, but not in WASP-expressing cells, such as macrophages. Chimera experiments using WASP and N-WASP revealed that N-WASP binds IcsA through its GTPase-binding domain that normally binds Cdc42 (Suzuki et al., 2002). Altogether, these studies suggest that IcsA acts as a functional mimic of Cdc42, releasing N-WASP from its auto-inhibitory conformation and facilitating the interaction of the nucleation-promoting domain (VCA) of N-WASP with the ARP2/3 complex (Kim et al., 2000). In addition to N-WASP, the passenger domain of IcsA has been shown to interact with IcsB (Ogawa et al., 2005), a T3SS effector protein required for *S. flexneri* dissemination (Allaoui et al., 1992; Ogawa et al., 2003). The exact function of IcsB is however complex and spans from inhibition of autophagy to disruption of the double membrane vacuole (Ogawa et al., 2005; Campbell-Valois et al., 2015). The exact significance of the IcsB-IcsA interaction in the context of *S. flexneri* dissemination remains therefore to be clarified. The passenger domain of IcsA was also shown to display potential sites for phosphorylation by cyclic AMP-dependent kinases (amino acid residues 754–760). The introduction of mutations preventing phosphorylation increased cell-to-cell spread, but only during the first 3 h of infection (d'Hauteville and Sansonetti, 1992).

In order to trigger N-WASP activation, *S. flexneri* also recruits to the bacterial surface the transducer of Cdc42-dependent actin assembly, TOCA-1 (Figure 2, step 1; Leung et al., 2008). TOCA-1 is member of the F-BAR family of membrane-deforming proteins that contribute to N-WASP activation (Ho et al., 2004). As opposed to N-WASP, the recruitment of TOCA-1 does not rely on IcsA, but requires the integrity of the transcriptional regulator VirB. This suggests a role for VirB-regulated virulence factors, including the T3SS, in TOCA-1 recruitment and further extend the notion that *S. flexneri* evolved complex functional mimicry in order to hijack the N-WASP/TOCA-1 machinery (Leung et al., 2008). N-WASP activation in the context of *S. flexneri* actin tail formation also relies on N-WASP tyrosine phosphorylation, which is positively regulated by the tyrosine kinases Abl and Btk, in fibroblasts and intestinal epithelial cells, respectively (Burton et al., 2005; Dragoi et al., 2013). In addition to the requirement for N-WASP and TOCA-1, biochemical and genetic studies indicated that additional cytoskeleton regulators, such as profilin, stimulate *S. flexneri* actin-based motility *in vitro* as well as *in vivo* (Loisel et al., 1999; Mimuro et al., 2000). Finally, N-WASP phosphorylation was shown to increase in a Btk-dependent manner in response to bacterial infection, suggesting that in addition to the components of the actin assembly machinery, *S. flexneri* manipulates signaling events that contribute to the efficiency of actin assembly at the bacterial pole, through unknown mechanisms (Dragoi et al., 2013).

## Protrusion formation in *S. Flexneri* dissemination

As bacteria displaying cytosolic motility reach the cell periphery, they deform the plasma membrane and form membrane protrusions. In order to lead to efficient cell-to-cell spread, the formed protrusions must project into adjacent cells (Figure 1, step 2). This is facilitated by the formation of E-cadherin-dependent cell-cell contacts in epithelial cells (Sansonetti et al., 1994). It was also suggested that tricellular junctions represent preferred sites of protrusion formation at the plasma membrane (Fukumatsu et al., 2012). In addition to structural components mediating cell-cell contacts, bacterial dissemination is also supported by connexin-dependent signaling events at the plasma membrane, through the release of signaling molecules, such as ATP (Tran Van Nhieu et al., 2003). There is also evidence for an active role played by the pathogen in the maintenance of cell-cell contacts supporting dissemination. This is exemplified by the observation that a mutant strain defective in the expression of OspE2, a type 3 secretion effector protein injected into host cells, leads to premature rounding up of infected cells, a reflection of cell-cell contact disruption (Miura et al., 2006; Kim et al., 2009). In order to form membrane protrusions, *S. flexneri* utilizes the forces developed by the actin cytoskeleton to deform the plasma membrane and project into adjacent cells. Similar to the mechanisms supporting actin tail formation in the cytosol, it is likely that actin polymerization in membrane protrusions relies on IcsA and the N-WASP/ARP2/3-dependent machinery (Figure 2, step 2). However, investigating the role of IcsA, N-WASP, and the ARP2/3 complex specifically in protrusions is difficult because disruption of the activity of these factors would primarily affect actin-based motility in the cytosol, and therefore prevent protrusions formation. Interestingly, a genetic screen for peptide insertions that interfere with IcsA function identified several positions (amino-acid residues 56, 193, 288, 312, and 502) at which peptide insertion hardly affected actin tail formation in the cytosol, and yet strongly affected plaque formation (May and Morona, 2008). The corresponding positions map to the region required for N-WASP recruitment and may be involved in the recruitment of host factors specifically required for protrusion formation, through a mechanism that remain to be further explored. In addition to the ARP2/3 complex, recent studies suggested the involvement of protrusions-specific and ARP2/3-independent actin nucleators in protrusion formation. In particular, Diaphanous-related formins have been identified as important determinants of protrusion formation in *S. flexneri* infected cells (Figure 2, step 2) (Heindl et al., 2010). Whether these formins contribute to actin polymerization at the bacterial pole, in concert with the ARP2/3 complex, or display additional functions in protrusions, needs to be further investigated. Future research should also examine whether similar to IcsA and the recruitment of the N-WASP/ARP2/3 machinery, pathogen-specific features regulate the recruitment/activity of formins in *S. flexneri* protrusions. In addition to cellular components supporting actin assembly, such as the ARP2/3 complex and formins, additional cytoskeleton factors contribute to protrusion formation, including myosins (Figure 2, step 2). Myosin X was recently found to localize to the plasma membrane surrounding *S. flexneri* protrusions and move along the bacterial sides (Bishai et al., 2013). Myosin X depletion resulted in the formation of shorter and wider protrusions, indicating a role for this protein in proper protrusion formation (Bishai et al., 2013). Structure function analyses suggested that Myosin X contributes to *S. flexneri* protrusion formation by bridging the actin cytoskeleton and the plasma membrane, and potentially transporting unknown cargoes within protrusions (Bishai et al., 2013). *S. flexneri* dissemination also relies on Myosin II and its regulator, the myosin light chain kinase MLCK (Rathman et al., 2000a; Lum and Morona, 2014). Interestingly, Myosin II is not required for the formation of *Listeria monocytogenes* protrusions (Rathman et al., 2000a), indicating a specific function for the Myosin II pathway in *S. flexneri* dissemination, which remains to be determined.

## Protrusion resolution in *S. Flexneri* dissemination

Recent live confocal microscopy of intestinal cells infected with *S. flexneri* revealed the formation of membrane protrusions that first undergo elongation and then transitioned into a membrane-bound compartment referred to as vacuole-like protrusions (Figure 1, step 3, VLP; Dragoi and Agaisse, 2015). VLPs display a continuous lining of the plasma membrane around the bacterium, but remain connected to the primary infected cells through a membranous tether. The tether results from the complete collapse of the protrusion neck, which likely reflects the collapse of the underlying actin cytoskeleton (Dragoi and Agaisse, 2014). The plasma membrane surrounding the protrusions formed by *S. flexneri* is highly enriched in phosphorylated tyrosine residues, suggesting a role for tyrosine kinase signaling in protrusions (Dragoi and Agaisse, 2014). Accordingly, treatment with the tyrosine kinase inhibitor Gleevec inhibited *S. flexneri* dissemination (Dragoi and Agaisse, 2014). Interestingly, tyrosine kinase signaling is supported by the cell polarity serine/threonine kinase STK11 through a mechanism that may involve the proper trafficking of receptor tyrosine kinases to cell-cell contacts (Dragoi and Agaisse, 2014). In addition to tyrosine phosphorylation, *S. flexneri* dissemination through protrusion collapse and VLP formation also requires the production of phosphatidylinositol-3-phosphate (PI(3)P) in protrusions, which relies on the class II phosphatidylinositol-3 kinase PIK3C2A (Dragoi and Agaisse, 2015). Interestingly, *Listeria monocytogenes* spread from cell to cell did not rely on tyrosine kinase and phosphoinositide signaling (Dragoi and Agaisse, 2015), highlighting the notion that *S. flexneri* and *L. monocytogenes* have evolved pathogen-specific mechanisms of bacterial dissemination (Kuehl et al., 2015). The first evidence of a role for the bacterial T3SS in the resolution of *S. flexneri* protrusions into vacuoles came from the isolation of a transposon in the *ipgC* gene, which encodes a molecular chaperone for IpaB and IpaC (Rathman et al., 2000b). The corresponding *ipgC* mutant grew normally in the cytosol of infected cells, where it displayed actin-based motility, but failed to spread from cell to cell. Electron microscopy studies revealed that the *ipgC* mutant resided in protrusions displaying several layers of membrane, as if the protrusions kept elongating and circling at sites of cell-cell contacts, instead of transitioning into vacuoles (Rathman et al., 2000b). Confirming the notion that the T3SS is required for proper dissemination, two independent studies demonstrated a role for MxiG, a structural component of the T3SS, in the resolution of protrusion into vacuoles (Allaoui et al., 1995; Kuehl et al., 2014). Moreover, MxiG was shown to be required for activation of tyrosine kinase signaling in *S. flexneri* protrusions (Kuehl et al., 2014). Altogether, these observations thus suggest that *S. flexneri* utilizes the T3SS to manipulate tyrosine kinase signaling and downstream signaling events, such as PI(3)P production, in order to mediate the collapse of the actin cytoskeleton in protrusions, which leads to VLP formation. Although the involvement of the T3SS in *S. flexneri* dissemination has been clearly established, the identity of the effector proteins potentially required for the manipulation of the signaling events observed in protrusions remains to be determined.

The mechanisms supporting the transition of VLPs into vacuoles are poorly understood (Figure 2, step 4). Live imaging studies suggested that the formation of genuine vacuoles occurs through the gradual disappearance of the membrane tether connecting VLPs to the primary infected cells (Dragoi and Agaisse, 2015). Interestingly, genetic depletion and pharmacological inhibition suggested a role for the Dynamin 2-dependent membrane scission machinery in *S. flexneri* dissemination (Fukumatsu et al., 2012). The resolution of the VLP membrane tether leading to vacuole formation may thus rely on the activity of Dynamin 2 in adjacent cells (Figure 2, step 4). Together with Dynamin 2, the class I PI3K, clathrin, epsin-1 and dynamin-2, but not AP-2, Dab2, and Eps15, were shown to be required for *S. flexneri* dissemination. These observations led the authors to propose a model in which the uptake of protrusions by adjacent cells occurs through a non-canonical form of endocytosis. Although clathrin has been shown to be recruited to large objects such as bacteria and be required for their uptake (Veiga and Cossart, 2006), it remains unclear how the known function of clathrin in coating small vesicles may assist in the internalization of large objects, including *S. flexneri* protrusions. Future research is therefore needed to clarify the role of Dynamin 2 and clathrin-mediated endocytosis in *S. flexneri* dissemination.

## Vacuole escape in *S. Flexneri* dissemination

The use of inducible systems to conditionally express components of the T3SS demonstrated that, in addition to its role in protrusions, the T3SS is also necessary for efficient escape from vacuoles (Figure 2, step 5; Page et al., 1999; Schuch et al., 1999; Kuehl et al., 2014). Electron microscopy studies first suggested that T3SS-defective mutants accumulate in the double membrane vacuoles formed during the dissemination process (Page et al., 1999; Schuch et al., 1999). These observations were recently confirmed by live imaging microscopy studies in which the tracking individual bacteria provided the unambiguous demonstration that a conditional mutant defective in the expression of the T3SS could not escape the vacuoles deriving from protrusion resolution (Kuehl et al., 2014). Interestingly, the use of a transcriptional reporter system to monitor the activity of the T3SS showed that T3 secretion is activated upon invasion, shut down in the cytosol, and re-activated in protrusions and vacuoles (Campbell-Valois et al., 2014). Moreover, the use of T3SS activity reporter in combination with bacterial mutants suggested that the VirA and IcsB mutants accumulate in double membrane vacuoles (Figure 2, step 5; Campbell-Valois et al., 2015). The function of IcsB is unknown, but several activities have been attributed to VirA, including inhibition of the small-GTPase Rab1 (Dong et al., 2012), and activation of the Calpain system (Bergounioux et al., 2012), through an unknown mechanism. The exact role of VirA in vacuole escape remains to be determined. As the single VirA and IcsB mutants hardly displayed plaque formation defects, and the double mutant did form smaller plaques (Campbell-Valois et al., 2015), it is likely that cooperative activities provided by various effector proteins contribute to double membrane vacuole escape. This may include the phosphoinositide phosphatase IpgD, which was shown to contribute to the escape from the primary vacuoles formed upon invasion (Mellouk et al., 2014). In addition to defining the complete set of effectors contributing to vacuole escape, the elucidation of the vacuole escape mechanisms will require the identification of the cellular components targeted by the T3SS effector proteins (Figure 2, step 5). Interestingly, a recent report established a link between the autophagy machinery and the repair of membrane damage caused by the activity of the T3SS in the primary vacuoles formed upon *Salmonella* infection (Kreibich et al., 2015). Given the proposed role of IcsB and VirA in countering autophagy (Ogawa et al., 2005; Dong et al., 2012; Campbell-Valois et al., 2015), it is tempting to speculate that vacuole escape partly occurs as a result of the failure of the autophagy machinery to repair the membrane of the vacuoles formed upon *S. flexneri* infection.

## Bacterial envelope biogenesis in *S. Flexneri* dissemination

*S. flexneri* dissemination relies on IcsA and the T3SS, two components of the bacterial envelope whose proper exposure on the bacterial surface is critical for their function. Not surprisingly, various determinants of bacterial envelope biogenesis have thus been identified as essential determinants of *S. flexneri* dissemination (Table 1).

**Table 1:** **The bacterial genes supporting ***Shigella flexneri*** dissemination**.

**Gene**	**Function**	**Defect**	**References**
*icsA*	N-WASP recruitment	Actin-based motility	Makino et al., 1986; Bernardini et al., 1989
*rfbBCAD*	LPS biosynthesis	IcsA polarity	Rajakumar et al., 1994
*galU*	LPS biosynthesis	IcsA polarity	Sandlin et al., 1995
*rfe*	LPS biosynthesis	IcsA polarity	Sandlin et al., 1995
*rol*	LPS biosynthesis	IcsA polarity	Morona et al., 1995
*ompT*	Protease	IcsA exposure	Nakata et al., 1993
*ompA*	?	IcsA exposure	Ambrosi et al., 2012
*degP*	Protease/Chaperone	IcsA exposure	Purdy et al., 2002
*skp*	Chaperone	IcsA exposure	Purdy et al., 2007
*surA*	Chaperone	IcsA exposure	Purdy et al., 2007
*phoN2*	Apyrase	IcsA exposure	Santapaola et al., 2006
*ompA*	?	IcsA exposure	Scribano et al., 2014
*ispA*	?	Septation	Mac Síomóin et al., 1996
*virK*	?	Envelope Biogenesis	Nakata et al., 1992; Sidik et al., 2014
*vpsC*	ABC Transporter	Envelope Biogenesis	Hong et al., 1998
*vacJ*	ABC Transporter	Envelope Biogenesis	Suzuki et al., 1994; Carpenter et al., 2014
*dsbA*	Periplasmic oxidoreductase	IpaB/IpaC secretion Protrusion resolution	Watarai et al., 1995; Yu et al., 2000
*dksA*	Transcription Factor	Hfq expression	Mogull et al., 2001
*hfq*	RNA binding	VirB expression?	Sharma and Payne, 2006
*pstS*	Phosphate binding	PhoB regulon mis-expression	Runyen-Janecky and Payne, 2002
*ipaB ipaC*	T3SS Translocases	Vacuole escape	Page et al., 1999; Schuch et al., 1999
*ipgC*	T3SS Chaperone	Protrusion resolution Vacuole escape?	Rathman et al., 2000b
*mxiE*	Transcription Factor	Effector protein expression	Kane et al., 2002
*mxiG*	Structural component	Protrusion resolution Vacuole escape	Allaoui et al., 1995; Kuehl et al., 2014
*virA*	Rab1 GAP, Calpain activation	Vacuole escape	Uchiya et al., 1995; Dong et al., 2012; Campbell-Valois et al., 2015
*icsB*	?	Vacuole escape	Allaoui et al., 1992; Ogawa et al., 2003, 2005; Campbell-Valois et al., 2015

*Relevant information includes the gene name, the function of the encoded protein, the observed defect in the context of S. flexneri dissemination, and the corresponding reference in the cited literature*.

### Establishment of IcsA polarity

The formation of actin tails at the bacterial pole relies on the establishment and maintenance of IcsA polarity (Goldberg et al., 1993). Although IcsA secretion relies on the circumferentially expressed Sec machinery, IcsA is only secreted at the bacterial pole (Brandon et al., 2003). Two regions in IcsA, termed region 1 (amino acid residues 56–104) and region 2 (amino acid residues 507–620) contribute to the polar localization of IcsA in the cytosol, prior to secretion (Charles et al., 2001). Genetic screens uncovered DnaK and FtsQ as regulators of IcsA cytoplasmic chaperoning and positioning at the bacterial poles, respectively (Fixen et al., 2012). In the periplasm, the protease/chaperone DegP functions in partnership with the chaperones Skp and SurA and mediates full exposure of the alpha domain of IcsA at the bacterial surface (Purdy et al., 2007).

### Maintenance of ICSA polarity

Early observations pointed to a role for LPS in *S. flexneri* dissemination (Okamura and Nakaya, 1977) and numerous genetic studies subsequently demonstrated the essential role of short O antigen assembly in plaque formation (Table 1). LPS-defective mutants are not able to form polar actin tails and instead polymerize actin circumferentially on the bacterial surface (Rajakumar et al., 1994; Sandlin et al., 1995; Van den Bosch et al., 1997). Interestingly, IcsA localized circumferentially in O antigen defective strains (Sandlin et al., 1995), suggesting that LPS supports the maintenance of IcsA at the bacterial pole by restricting its lateral diffusion. In addition to LPS, the serine protease IcsP was proposed to contribute to the maintenance of IcsA polarity by releasing the N-terminal domain from the bacterial sides (Shere et al., 1997). However, compared to wild-type bacteria, the *icsP* mutant was indeed more efficient at moving in the cytosol and spreading from cell to cell, indicating that the IcsP-mediated cleavage of IcsA is in fact detrimental to *S. flexneri* dissemination (Shere et al., 1997). It is noteworthy that outer membrane proteins detrimental to IcsA expression in *E. coli*, such as OmpT, are no longer encoded in *S. flexneri* spp. genomes (Nakata et al., 1993). Deletion of “anti-virulence” genes has been proposed to be one of the major determinants in the evolution of *S. flexneri* pathogenic properties, including the acquisition of IcsA-mediated actin-based motility (Bliven and Maurelli, 2012).

### Assembly of the T3SS

VpsC was identified as a determinant of *S. flexneri* dissemination in a screen for bacterial factors required for plaque formation (Hong et al., 1998). VpsC is part of the Vps/VacJ transporter system and a similar plaque formation defect was originally observed for the *vacJ* mutant (Suzuki et al., 1994). The Vps/VacJ system has been proposed to regulate the levels of phospholipids in the outer leaflet of the outer membrane in *E. coli* (Malinverni and Silhavy, 2009). However, enzymatic manipulation of phospholipid composition aiming at restoring normal levels of phospholipids in the outer membrane, failed to correct the plaque formation defect observed with the *vpsC* mutant (Hong et al., 1998). Interestingly, the VpsC mutant secretes elevated levels of the virulence factors IcsA, IpaB, IpaC, and IpaD (Hong et al., 1998) and a similar hypersecretion phenotype was reported recently for the *virK* mutant (Sidik et al., 2014). The exact function of VirK is unknown, but hypersecretion is apparently due to an increase in the release of outer membrane vesicles (OMVs). Moreover, a similar increase in OMV release was reported for a mutant in the periplasmic chaperone DegP. As both *virK* and *degP* showed genetic interactions with *mxiD*, a structural component of T3SS, it was proposed that VirK and DegP may relieve the periplasmic stress associated with the assembly of the T3SS (Sidik et al., 2014). Also required for the proper assembly of the T3SS is the periplasmic thiol:disulfide oxidoreductase DsbA. Interestingly, the *dbsA* mutant displayed normal actin-based motility, but failed to resolve membrane protrusions into vacuoles (Yu et al., 2000). This defect was associated with defective secretion of the translocases, IpaB and IpaC, in protrusions (Watarai et al., 1995; Yu et al., 2000). Since the *dbsA* mutant did not display any T3SS-dependent invasion defects, it is likely that the activity of DbsA is specifically required for the assembly of the T3SS in protrusions. These observations point to the fact that we know very little about the mechanisms supporting the post-invasion assembly of the T3SS in various cellular compartments, including the cytosol, protrusions and vacuoles.

## Bacterial adaptation in *S. Flexneri* dissemination

*S. flexneri* is an intracellular pathogen that resides in the cytosol of infected cells, but also encounters various membrane-bound compartments upon dissemination, including membrane protrusions and vacuoles in which the bacteria spend an average of ~15 and ~30 min, respectively (Dragoi and Agaisse, 2015). *S. flexneri* may thus need to adapt to various cellular environments in order to disseminate successfully. Accordingly, a screen for bacterial genes induced in the intracellular environment led to the identification of genes required for phosphate acquisition (*pstS*), sugar import (*uphT*) and genes activated in response to iron depletion (*sitA*; Runyen-Janecky and Payne, 2002). Although the *uphT* and *sitA* mutant strains formed normal plaques, mutants in the Pst system formed smaller plaque compared to wild type (Runyen-Janecky and Payne, 2002). The spreading defect was not related to PhoA-mediated phosphate transport, but involved the de-regulation of the PhoB regulon, which did not impact IcsA polarity or bacterial growth, at least during the first 3 h of infection (Runyen-Janecky et al., 2005). Why the expression of PhoB-regulated genes negatively impacted *S. flexneri* dissemination is unclear, but it indicates the importance of proper regulation of bacterial gene expression profiles in the dissemination process. In addition to the main transcriptional regulators of *S. flexneri* virulence, VirB and VirF, the transcriptional activator MxiE regulates the expression of a subset of T3SS effector proteins (Kane et al., 2002; Mavris et al., 2002). MxiE is regulated by the activity of the T3SS through a complex mechanism involving the chaperone IpgC, acting as MxiE co-activator, and the secreted effector OspD1, acting as MxiE inhibitor (Parsot et al., 2005). When the T3SS is not active, the interaction of OspD1 with MxiE prevents transcriptional activation of the MxiE-regulated genes. As the T3SS system gets activated in protrusions or vacuoles, probably upon insertion of the translocases into the plasma membrane, the secretion of OspD1 frees up MxiE, which triggers the transcriptional activation of MxiE-regulated genes. As the *mxiE* mutant displays a defect in cell-to-cell spread (Kane et al., 2002), it is likely that a set of MxiE-regulated effectors proteins, contributes to *S. flexneri* dissemination. Recent work pointed to a role for the MxiE-regulated effector protein VirA in vacuole escape (Campbell-Valois et al., 2015). Future research should therefore systematically explore the potential role of MxiE-regulated effectors proteins in *S. flexneri* dissemination.

## Concluding remarks

In the past three decades, we have made tremendous advances in our understanding of the mechanisms supporting *S. flexneri* dissemination through cell-to-cell spread. Genetic investigations have revealed two critical bacterial determinants of dissemination: the auto-transporter IcsA and the T3SS.

The role of IcsA in acquisition of actin-based motility in the cytosol, though interaction with the N-WASP/ARP2/3 actin assembly machinery, has been extensively investigated and is now fairly well understood. By contrast, the mechanisms supporting actin assembly in membrane protrusions are still poorly understood. In particular, the exact role of cytoskeleton factors, such as formins and myosins, and potential bacterial factors, specifically required for membrane protrusion formation need further attention.

Genetic investigations have demonstrated the requirement of the T3SS for efficient *S. flexneri* dissemination. The determination of the exact mechanisms supporting T3SS-dependent dissemination will not only require the identification and characterization of the T3SS effector proteins potentially involved, but also the identification and characterization of the cellular processes targeted by the T3SS effector proteins. This relates to T3SS-dependent actin-based motility, the complex protrusion-to-VLP-to-vacuole transition, and the escape from double membrane vacuoles. Given the multiple roles of the T3SS, different sets of effectors may act cooperatively in different cellular compartments, perhaps suggesting the existence of uncharacterized regulatory mechanisms controlling the activity of the T3SS in space and time during the course of dissemination.

Beyond the IcsA- and T3SS-dependent mechanisms mediating cytosolic motility and cell-to-cell spread, it is unclear whether specific mechanisms support bacterial fitness in the various cellular compartments encountered by the pathogen during dissemination, including the cytosol, membrane protrusions and double membrane vacuoles. How the bacteria may sense, respond and adapt to these various cellular environments remains poorly understood and may represent critical aspects of the dissemination process. Finally, although the dissemination process is central to the extensive damage inflicted to the intestinal epithelium during infection, the exact role of cell-to-cell spread in *S. flexneri* pathogenesis is still unclear. It is generally admitted that the destruction of the epithelial mucosa results from the infiltration of immune cells in response to pro-inflammatory molecules produced by infected epithelial cells. Although the dissemination process certainly contributes to the magnitude of the inflammatory response by enlarging the numbers of epithelial cells producing pro-inflammatory molecules, the impact of cell-to-cell spread on epithelial homeostasis may be far more complex than currently understood.

In conclusion, three decades of research on *S. flexneri* actin-based motility and cell-to-cell spread have contributed to our current understanding of the molecular and cellular mechanism supporting *S. flexneri* dissemination. It is noteworthy that, in addition to *S. flexneri*, actin-based motility and cell-to-cell spread are essential aspects of the infectious cycle of various intracellular pathogens, including *Listeria monocytogenes, Rickettsia* spp. and *Burkholderia* spp. Recent studies have highlighted the notion that, although relying on common strategies of intracellular motility based on the actin cytoskeleton, *S. flexneri* and *L. monocytogenes* have in fact evolved strikingly different mechanisms of cell-to-cell spread (Talman et al., 2014; Dragoi and Agaisse, 2015; Kuehl et al., 2015). Further comparative analyses of the mechanisms supporting *S. flexneri, L. monocytogenes, Rickettsia* spp. and *Burkholderia* spp. spread from cell to cell may thus reveal an astonishing diversity of mechanisms supporting bacterial dissemination.

## Author contributions

The author confirms being the sole contributor of this work and approved it for publication.

### Conflict of interest statement

The author declares that the research was conducted in the absence of any commercial or financial relationships that could be construed as a potential conflict of interest.
